# Eco-Friendly Extraction, Structure, and Gel Properties of ι-Carrageenan Extracted Using Ca(OH)_2_

**DOI:** 10.3390/md20070419

**Published:** 2022-06-27

**Authors:** Feng Jiang, Yao Liu, Qiong Xiao, Fuquan Chen, Huifen Weng, Jun Chen, Yonghui Zhang, Anfeng Xiao

**Affiliations:** 1 College of Food and Biological Engineering, Jimei University, Xiamen 361021, China; 202011832019@jmu.edu.cn (F.J.); liuyao0902@foxmail.com (Y.L.); xiaoqiong129@jmu.edu.cn (Q.X.); fqchenhy0109@jmu.edu.cn (F.C.); wenghuifen@jmu.edu.cn (H.W.); chenjun@jmu.edu.cn (J.C.); 2 National R&D Center for Red Alga Processing Technology, Xiamen 361021, China; 3 Fujian Provincial Engineering Technology Research Center of Marine Functional Food, Xiamen 361021, China; 4 Xiamen Key Laboratory of Marine Functional Food, Xiamen 361021, China

**Keywords:** ι-carrageenan, calcium hydroxide, cleaner production, gel characteristics

## Abstract

An eco-friendly method for ι-carrageenan extraction from seaweed *Eucheuma denticulatum* through boiling and using a low concentration of Ca(OH)_2_ is reported. Compared to the traditional method of ι-carrageenan extraction using NaOH, the reported method using Ca(OH)_2_ had the advantages of using 93.3% less alkali and 86.8% less water, having a 25.0% shorter total extraction time, a 17.6% higher yield, and a 43.3% higher gel strength of the product. In addition, we evaluated the gel properties and structures of ι-carrageenan products extracted by Ca(OH)_2_ (Ca-IC) and NaOH (Na-IC). The Fourier transform infrared spectroscopy results showed that the structures of Ca-IC and Na-IC did not change remarkably. The results of the thermogravimetric analysis and differential scanning calorimetry showed that Ca-IC had the same thermal stability as Na-IC. The results of the textural analysis showed that Ca-IC had a higher hardness and better chewiness compared to Na-IC. Rheological results indicated that Ca-IC and Na-IC exhibited shear-thinning and non-Newtonian fluid properties, whereas the viscosity of Ca-IC was less than that of Na-IC. In conclusion, this new method of ι-carrageenan extraction using Ca-IC is markedly better and yields higher quality carrageenan than the conventional method of using Na-IC.

## 1. Introduction

Carrageenan is a sulfated linear polysaccharide composed of galactose and sulfate groups distributed in polymer chains. Carrageenan is soluble in hot water and can form thermally reversible gels with K^+^ or Ca^2+^ [1]. The raw material of carrageenan is red algae, and different types of carrageenan are obtained from different types of seaweeds, such as kappa, iota, and lambda [2]. Among these types, ι-carrageenan, which is an alternating disaccharide unit consisting of (1–3)-linked α-d-galactose-4-sulfate and (1–4)-linked 3,6-anhydro-β-d-galactose-2-sulfate, is extracted from *Eucheuma denticulatum* (*E. denticulatum*) [3,4]. ι-carrageenan has been widely used in the food, medical, and material industries because of its gelling properties, stability, and synergistic effects. For example, the good stability of ι-carrageenan makes the addition of ι-carrageenan to ice cream reduce the growth of ice crystals [5]. The gelling properties of ι-carrageenan and the low toxicity of the digestive products allow the preparation of novel ι-carrageenan/gelatin hydrogel capsules [6]. Furthermore, in the medical field, ι-carrageenan can be used as an effective inhibitor of the influenza A viral infection, most importantly used in vitro to suppress the 2009 flu pandemic and oseltamivir-resistant H1N1 influenza strains [7].

Currently, high-quality carrageenan has a high gel strength, a low sulfation, and a high 3,6-anhydro-d-galactose (3,6-AG) content. Studies found that the use of a high concentration of alkali can reduce the sulfate content of carrageenan and increase the 3,6-AG content, thus improving the gel strength of carrageenan [8,9]. Therefore, the extraction of carrageenan is usually performed by alkali treatment of the raw material followed by washing, alkali cooking, alcohol precipitation, drying, and crushing. However, the carrageenan yield obtained by this method is low and large amounts of alkali and water need to be used, thus increasing the cost of treating wastewater exponentially [10]. To improve the extraction process, researchers proposed various eco-friendly methods, including microwave- [11,12], ultrasound- [13], and enzyme-assisted extraction [14,15]. Microwave- and ultrasound-assisted extraction methods have remarkably improved production efficiency, but large-scale production is not possible due to several factors, such as production cost. The enzyme-assisted extraction or direct extraction of carrageenan by using enzymes has received attention and remarkably improved the production efficiency of agar [16,17,18]. Enzyme-assisted extraction reduces the amount of alkali and improves yield, but the quality of the produced colloids is not remarkable because the enzyme is easily inactivated and the stability of the enzyme cannot be guaranteed in practical production. In the extraction of ι-carrageenan, the most commonly used method is the high-concentration alkali cooking method, which consumes large amounts of alkali and water and produces large amounts of wastewater. Therefore, a simple and environmentally friendly extraction method should be developed and applied in practical production.

The current industrial production process of ι-carrageenan is still the traditional alkali treatment process which has complex steps; consumes large amounts of alkali, water, and alcohol; and requires a large investment in wastewater treatment. Similarly, the problem exists in the industrial production of κ-carrageenan. A new process for the extraction of κ-carrageenan with Ca(OH)_2_ and CO_2_ is established in our previous study to solve the existing problem [19]. This method extracts carrageenan by adding Ca(OH)_2_ at high temperatures and neutralizing the sample by adding CO_2_. Most of the CaCO_3_ precipitates can be removed by filtration, whereas the residual CaCO_3_ precipitate can be removed by conversion to Ca(HCO_3_)_2_ by excess CO_2_ aeration during carrageenan extrusion and dehydration. The Ca(OH)_2_ method remarkably reduces the amount of water and alkali and obtains better κ-carrageenan compared to the traditional process. Therefore, a production process for the treatment of ι-carrageenan with Ca(OH)_2_ should be developed by borrowing from the production process of Ca(OH)_2_ for the extraction of κ-carrageenan. 

Different from κ-carrageenan, ι-carrageenan bears two sulfate groups, which makes the presence of Ca^2+^ promote the aggregation of ι-carrageenan molecules [20,21] and prevent the complete separation of ι-carrageenan molecules from the algal cell wall, ultimately leading to unstable yield and quality. Therefore, we separated ι-carrageenan molecules from seaweed impurities by pretreatment with boiling water followed by alkali treatment to obtain a high yield. In addition, Ca^2+^ can balance the sulfate groups on ι-carrageenan molecular chains [21]. Thus, sulfate groups are easily removed, and the quality of ι-carrageenan is improved. In the presence of Ca^2+^, a pair of two sulfate groups on adjacent helices of the carrageenan molecule form a bridge, which complicates the molecular arrangement. This phenomenon may lead to changes in the gel properties of carrageenan [22]. For this purpose, we attempt to extract ι-carrageenan by Ca(OH)_2_ (Ca-IC) instead of NaOH (Na-IC) and compare the two processes in terms of extraction process, gel properties, and basic components of products. Then, we use Fourier transform infrared (FTIR) spectroscopy and thermal analysis (TG-DTG and differential scanning calorimetry [DSC]) to characterize the structure and compare the gel properties of the two types of ι-carrageenan by using a texture profile analysis (TPA) and a rheological analysis to provide a theoretical basis for practical production.

## 2. Results and Discussion

### 2.1. Comparison of the Extraction of ι-Carrageenan by Using NaOH and Ca(OH)_2_

The industrial process of carrageenan extraction uses high concentrations of alkali to pretreat seaweed at low temperatures and obtain high-purity and high-quality carrageenan. Carrageenan is locked in the cell wall matrix, and alkali treatment leads to the rupture of the cell membrane, where alkali- and cold water-soluble substances are subsequently removed during washing [23]. At the same time, unfinished 6-sulfated molecules can be converted into 3,6-AG [24], which improves the gel strength of carrageenan. In this experiment, the direct disruption of the cell wall by boiling is used to leach the carrageenan, and the full exposure of sulfate groups on the carrageenan molecular chain in the solution state [25] can be removed with low concentrations of alkali. Impurities are removed during filtration and alcohol sedimentation.

**Figure 1:**
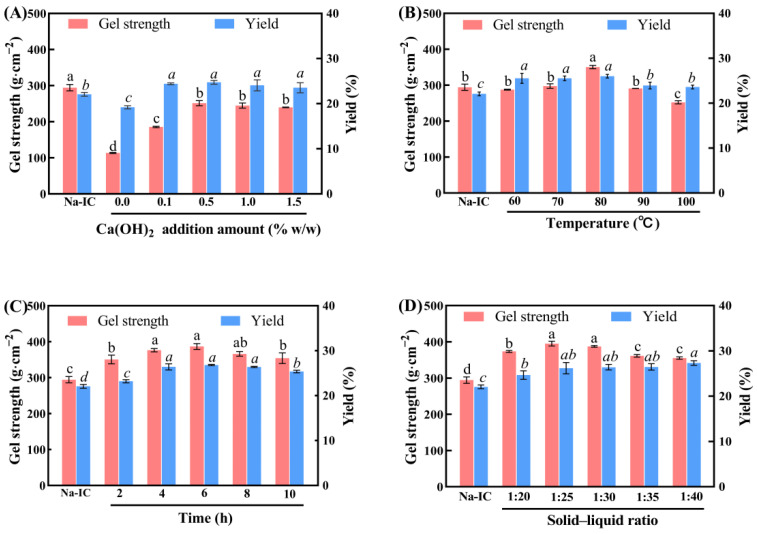
Gel strength and yield of ι-carrageenan. (**A**) Ca(OH)_2_ addition amount (0, 0.1%, 0.5%, 1.0%, and 1.5%, g·g^−1^). (**B**) Treatment temperature (60 °C, 70 °C, 80 °C, 90 °C, and 100 °C). (**C**) Alkali treatment time (2, 4, 6, 8, and 10 h). (**D**) Solid–liquid ratio (1:20, 1:25, 1:30, 1:35, and 1:40). Different letters denote significant difference (*p* < 0.05), and bars represent standard deviations (*n* = 3).

Gel strength and yield are two important factors in the production of ι-carrageenan. Therefore, the process of ι-carrageenan extraction by the Ca(OH)_2_ method is optimized using gel strength and yield as factors. The effect of different extraction factors on the gel strength and yield of ι-carrageenan is shown in Figure 1. The appropriate amount of alkali can remove the sulfate groups from carrageenan molecules and improve the gel strength of carrageenan [26]. Thus, the additional amount of Ca(OH)_2_ was 0.5% (*w*/*w*). Compared to the traditional industrial extraction of ι-carrageenan, the new method used remarkably less amount of alkali [9]. The extraction temperature and extraction time were beneficial in improving the quality of carrageenan, but the extremely high temperature used and the extremely long extraction time caused the degradation of the carrageenan. The extraction temperature and time were 80 °C and 4 h, respectively [27]. Finally, considering the product quality and water-saving effect, 1:25 was determined as the optimal solid–liquid ratio.

Under optimized conditions, the 500 L test was performed using Ca(OH)_2_ extraction method, whereas the NaOH extraction of the same scale was performed as a control. The gel strength and yield (337.1 ± 25.9 g·cm^−2^, 24.7% ± 2.5%) of Ca-IC obtained under this process were higher by 43.3% and 17.6%, respectively, than those of Na-IC (235.2 ± 12.2 g·cm^−2^, 21.0% ± 0.5%, respectively).

**Figure 2:**
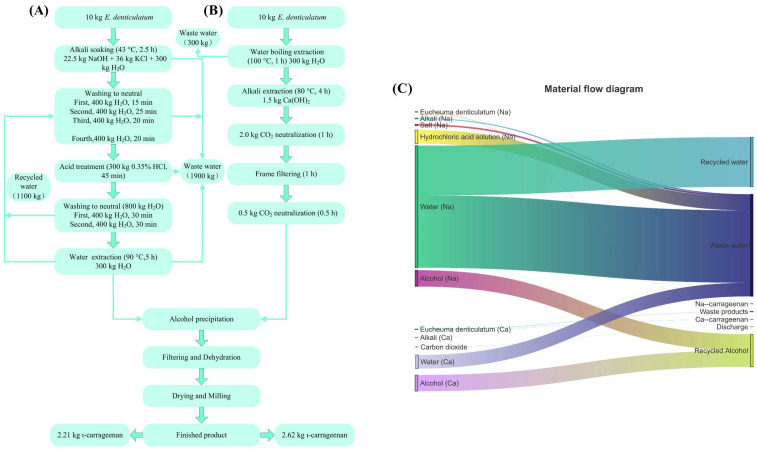
Process flow and material consumption for the production of ι-carrageenan by two methods in pilot-scale production: (**A**) NaOH and (**B**) Ca(OH)_2_ methods. (**C**) Material flow diagram of ι-carrageenan extraction by NaOH and Ca(OH)_2_.

The extraction of ι-carrageenan by NaOH and Ca(OH)_2_ methods is shown in Figure 2. On the basis of the consumption of 10 kg of raw material, the Ca(OH)_2_ method had a relatively simple production process of ι-carrageenan and a shortened production cycle by 25.0% compared to the NaOH method. The total water consumption of the Ca(OH)_2_ method was only 250 kg, which was 86.8% less than that of the NaOH method (1900 kg). The amount of alkali consumed by the Ca(OH)_2_ method (1.5 kg) was also much less than that consumed by the NaOH method (22.5 kg). This finding was because the extraction of ι-carrageenan by the Ca(OH)_2_ method with alkali pretreatment was carried out in a solution, and the contact between carrageenan and alkali within the solution state was sufficient. Additionally, only a very small amount of alkali was needed to remove enough sulfate, and the reduction of alkali dosage simultaneously reduced the amount of water needed for cleaning. Meanwhile, Figure 2C showed that the waste generated by the NaOH method was complex in composition and difficult to treat. In contrast, the wastewater generated by the Ca(OH)_2_ method could be directly recycled after simple neutralization and filtration, and the waste generated consisted of seaweed residue, a small amount of alkali, and a small amount of CaCO_3_, which was expected to be a raw material for the production of carrageenan oligosaccharides. Therefore, the Ca(OH)_2_ method was an efficient and water-saving process for the extraction of ι-carrageenan.

### 2.2. Determination of Physicochemical Properties

Sulfate and 3,6-AG contents are the two main factors affecting the gel strength of carrageenan. When the extracted carrageenan has a low sulfate content or a high 3,6-AG content, the gel strength of the colloid is high [9]. As shown in Table 1, the sulfate contents of Na-IC and Ca-IC were not significantly different, whereas the 3,6-AG content of Ca-IC was significantly increased by 13.6% compared to that of Na-IC (*p* < 0.05). This finding might be due to the interactive forces between Ca ions and sulfate groups on the molecular weight of ι-carrageenan [28], and the formation of CaSO_4_ residues during extraction increased the total sulfate content of Ca-IC. Therefore, the Ca(OH)_2_ method showed improved desulfurization, which facilitated the formation of 3,6-AG and resulted in high gel strength.

The whiteness of carrageenan indicates a quite complete pigment removal during the extraction process and directly affects the product quality, performance, and usage. The whiteness of Ca-IC (56.7% ± 0.7%) was higher than that of Na-IC (48.6% ± 0.4%), indicating that the extraction process by the Ca(OH)_2_ method was more favorable for pigment removal than that by the NaOH method.

The viscosity of Ca-IC (50.8 ± 1.5 cP) was significantly lower than that of Na-IC (25.4 ± 1.2 cP) (*p* < 0.05). The viscosity of the gel decreased with increased cooking time and temperature [29]. The viscosity of Ca-IC was significantly lower than that of Na-IC, which was probably due to the degradation of polysaccharides and decrease in molecular weight caused by the high temperature and prolonged treatment, whereas a decrease in molecular weight caused a decrease in viscosity.

### 2.3. Elemental Analysis

**Figure 3:**
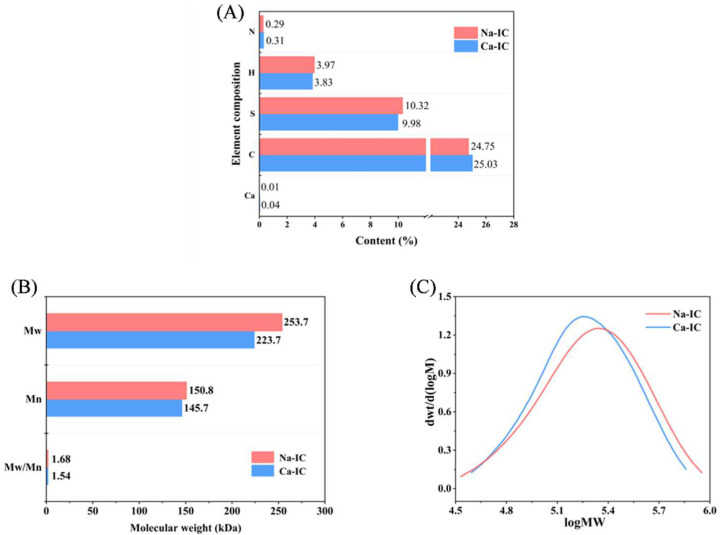
(**A**) Elemental analysis. (**B**) Molecular weight. (**C**) Molecular weight distribution.

Figure 3A depicts the contents of C, H, N, and S in Ca-IC and Na-IC. Carrageenan is a polysaccharide containing sulfate groups and has high contents of C and S elements. In addition, the S content of Ca-IC was similar to that of Na-IC, which was consistent with the sulfate content data reported in Table 1. The low content of N in both samples suggested few protein impurities.

Ca-IC (0.04%) had a slightly higher Ca content than Na-IC (0.01%), which might be due to residual ions in the extraction step. The Ca content of Ca-IC was only 0.04%, and Bui [30] found that when the concentration of CaCl_2_ was higher than 20 mM, the gel strength of carrageenan was not affected. In this experiment, 0.2% (*w*/*w*) CaCl_2_ was required to assess the gel properties of Ca-IC and Na-IC. Thus, the difference in their properties was determined by their structure and not markedly related to the residual Ca content [31].

### 2.4. Molecular Weight

Figure 3B,C show the molecular weight of carrageenan. The polydispersity index of Ca-IC was 1.54, which was slightly lower compared to that of Na-IC (1.68), and the molecular weight of Ca-IC (223.7 kD) was slightly lower than that of Na-IC (253.7 kD). This finding might be due to the degradation of carrageenan molecules caused by prolonged high temperatures [27], whereas the low molecular weight might also be one of the reasons for the low viscosity of Ca-IC. The gel strength of carrageenan weakened with decreasing molecular weight, and the Ca-IC gel strength was higher than the Na-IC gel strength, indicating that the 3,6-AG content was the most critical element affecting gel strength.

### 2.5. FTIR Spectroscopy

**Figure 4:**
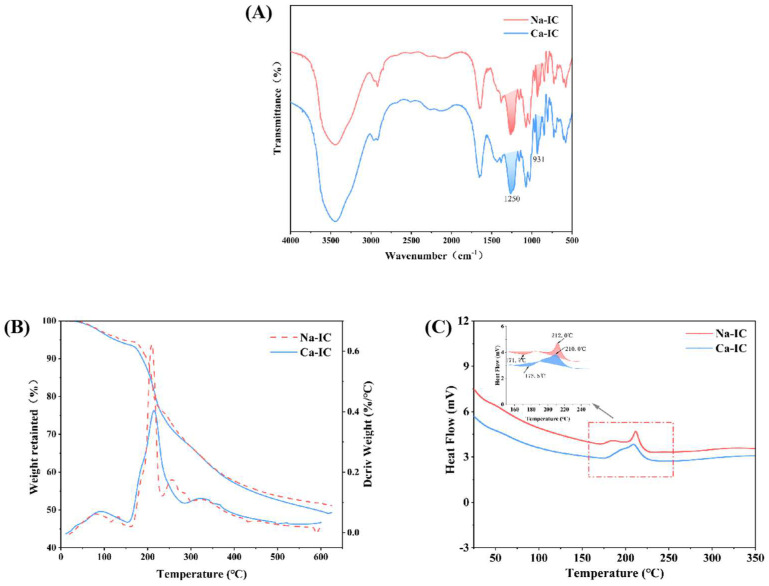
(**A**) FTIR spectra, (**B**) TG-DTG curves, and (**C**) DSC curves of Ca-IC and Na-IC.

Figure 4A shows the FTIR spectra of Ca-IC and Na-IC. The broad peak at 3400 cm^−1^ was caused by the stretching vibration of the hydroxyl group (O–H). The absorption peaks at 2900 and 1650 cm^−1^ were caused by the stretching vibrations of C-H and bound water, respectively. The absorption peak at 1250 cm^−1^ was caused by the asymmetric stretching vibration of the oxygen atom, which confirmed the existence of the sulfate group. The absorption peak at 931 cm^−1^ was attributed to 3,6-AG. Vibrations were also present at 1068 cm^−1^ due to the galactose backbone. The spectra showed no great difference in the absorption peak at 1250 cm^−1^ for Ca-IC compared to that of Na-IC, whereas the absorption peak at 931 cm^−1^ was stronger, indicating that the total sulfate content of Ca-IC was not different compared to Na-IC. The content of 3,6-AG was higher. Sulfate groups were present at C2 and C4 positions, which could be proven by the absorption peaks at 805 and 840 cm^−1^ [32]. The significant peaks and structural information of the two carrageenans are shown in Figures S1 and S2 (Supplementary Material).

### 2.6. Thermal Analysis

Figure 4B,C show the TG-DTG and DSC curves of Ca-IC and Na-IC. The weight loss process of carrageenan was summarized in two stages. In the first stage, the weight loss was due to the volatilization of free water within the carrageenan molecules, and no obvious difference was observed in the weight loss of Ca-IC compared to that of Na-IC. In the second stage, weight loss was due to the decomposition of ι-carrageenan [33], in which galactose hydroxyl groups were rapidly dehydrated and decomposed; the C-H, C-O, and C-C bonds were broken; and the main chain was interrupted. The DTG results showed that the decomposition temperatures of Na-IC and Ca-IC were 161 °C and 160 °C, respectively, which indicated no difference in the thermal stability of Na-IC and Ca-IC powders.

Figure 4C shows the DSC curves. Ca-IC and Na-IC had evident heat absorption and exothermic peaks. Ca-IC had higher heat absorption peak temperature (176.5 °C) than Na-IC (171.5 °C), but both samples had the same heat absorption peak area, which was caused by the evaporation of residual water molecules. Na-IC and Ca-IC had the same magnitude of heat absorption peaks, indicating the same evaporation of residual water molecules. This finding was consistent with the results of weight loss in the first stage of the TG-DTG curve. No obvious change was observed in the exothermic peak temperature (210.0 °C) of Ca-IC compared to that of Na-IC (212.0 °C). This finding also indicated that Na-IC and Ca-IC powders had the same thermal stability [34].

### 2.7. TPA

**Figure 5:**
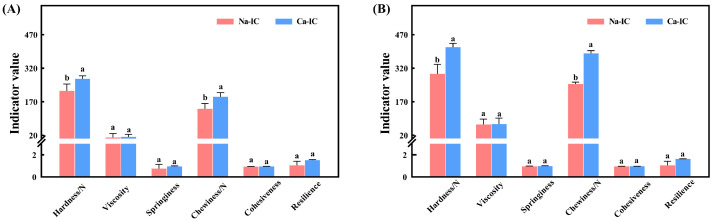
Texture of different concentrations of Ca-IC and Na-IC: (**A**) 2% (*w*/*v*) and (**B**) 3% (*w*/*v*). Different letters denote significant difference (*p* < 0.05), and bars represent standard deviations (*n* = 3).

The mechanical properties of carrageenan can be expressed by “hardness”, “cohesiveness”, “chewiness”, “resilience”, “viscosity”, and “springiness” [35]. The gel texture properties of Ca-IC and Na-IC at two concentrations were determined using a texture analyzer (Figure 5). Notably, the TPA parameters of ι-carrageenan obtained by the two extraction methods were consistent at 2% and 3% concentrations. The textural properties of ι-carrageenan at different concentrations showed a consistent trend. The chewiness of Ca-IC was higher compared to that of Na-IC, which was beneficial for the application in food. The hardness of Ca-IC was significantly higher than that of Na-IC (*p* < 0.05), which was consistent with gel strength results [36] and might be related to the denser gel network structure of the former [37]. Viscosity, springiness, cohesiveness, and resilience of Ca-IC were not significantly different from those of Na-IC (*p* > 0.05). The gel mechanical properties of Ca-IC were better than those of Na-IC.

### 2.8. Rheological Characterization

#### 2.8.1. Steady Rheological Testing

**Figure 6:**
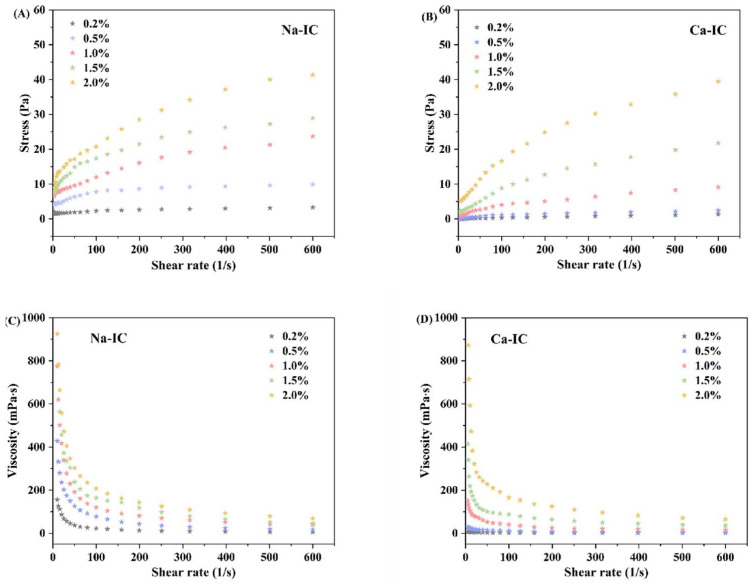
Steady rheological testing. Effect of concentration on the shear rate and viscosity of (**A**,**C**) Na-IC and (**B**,**D**) Ca-IC solutions.

As shown in Figure 6, the apparent viscosity and shear stress of Na-IC and Ca-IC solutions increased with increasing concentration at the same shear rate. All samples exhibited shear-thinning behavior, indicating that all samples had non-Newtonian pseudoplastic fluid properties [38]. This finding was because the molecular chains of carrageenan in the solution became oriented in the flow direction as the shear rate increased, resulting in a relative decrease in intermolecular forces [39,40]. In addition, as shown in Figure 6D, shear-thinning was only evident at Ca-IC concentrations of 1.0% or higher, which was related to the lower viscosity of Ca-IC compared to that of Na-IC. Subsequently, we fitted the flow curves of ι-carrageenan extracted by different processes to a power law model [41] (Table 2), and the obtained *R^2^* values were all greater than 0.99, indicating that the flow curves of ι-carrageenan solutions were well correlated with the model. By comparison, the value of n for the flow index of Ca-IC was larger than that of Na-IC, indicating that the solution of Ca-IC had less resistance to flow and was more likely to exhibit the properties of a Newtonian fluid under the same concentration of both carrageenan solutions compared to that of Na-IC.

#### 2.8.2. Frequency Sweep Test

**Figure 7:**
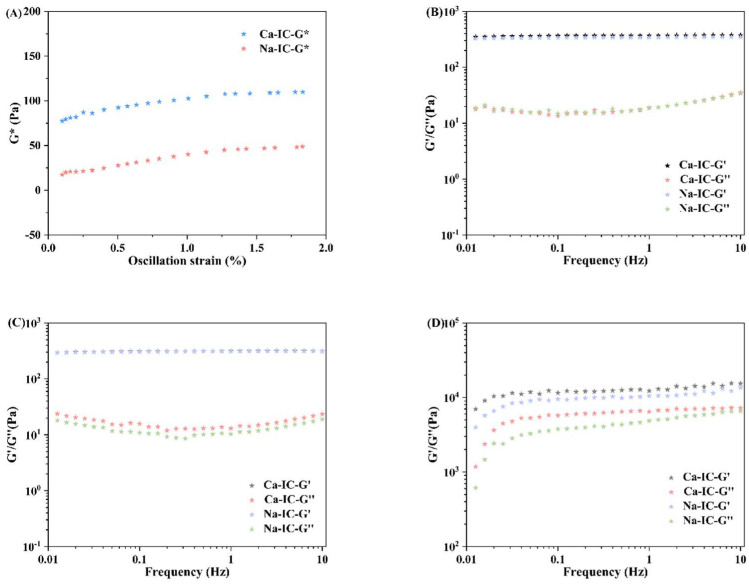
(**A**) Relationship between G* and oscillatory strain for Ca-IC and Na-IC solutions. Frequency sweep curves of Na-IC and Ca-IC at (**B**) 4 °C, (**C**) 25 °C, and (**D**) 55 °C.

Viscoelasticity is usually expressed by elastic (G′) and viscous (G”) moduli. When G′ is higher than G”, the viscoelastic material reflects elastic properties, whereas when G” is higher than G′, the viscoelastic material reflects viscous properties [42]. The linear viscoelastic region is the region where the composite modulus G* (G* = G′ + iG”) does not vary with oscillatory strain or stress. The relationship between the composite modulus and the oscillatory strain for the ι-carrageenan solution system extracted by different processes is shown in Figure 7A. Ca-IC and Na-IC showed linear viscoelastic regions at strains around 1.0%. Therefore, strains were fixed at 1.0% for the subsequent experiments.

Figure 7B–D show the viscoelastic modulus of carrageenan at different scanning frequencies and temperatures (4 °C, 25 °C, and 55 °C). At 4 °C and 25 °C, the carrageenan exhibited a clear elastic behavior with increasing frequency, with G′ always larger than G”. The G′ and G” values of Ca-IC were not remarkably different from those of Na-IC, indicating that the viscoelasticity of the two carrageenan gels was consistent. Figure 7D shows that the G′ and G” values of Ca-IC were higher than those of Na-IC at 55 °C. Therefore, Ca-IC had better viscoelasticity than Na-IC in the gel state [43].

#### 2.8.3. Temperature Sweep Test

**Figure 8:**
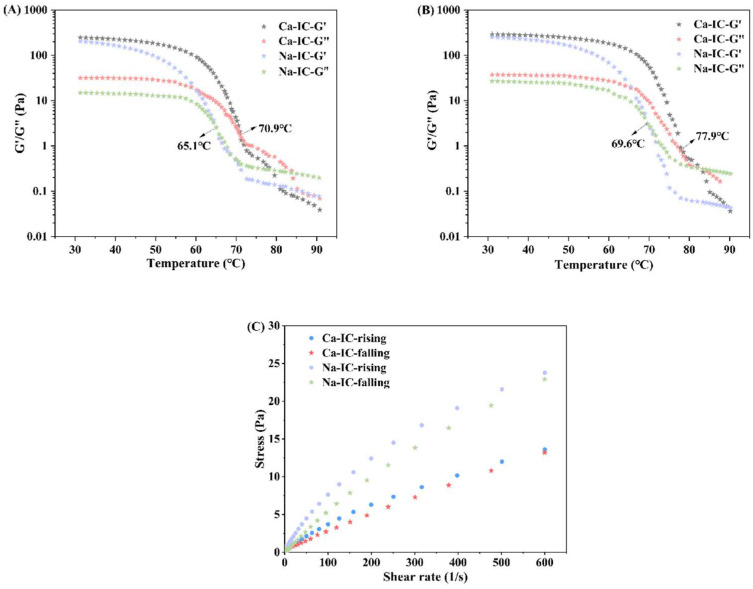
Dynamic rheological testing. Temperature dependence of storage (G′) and loss (G”) moduli of Ca-IC and Na-IC: (**A**) cooling and (**B**) heating processes. (**C**) Thixotropy curves of Na-IC and Ca-IC.

Figure 8A,B show the behavior of G′ versus G” with temperature for Ca-IC and Na-IC solutions. ι-Carrageenan showed the same trend during heating as during cooling. Figure 8A shows the cooling process. As the temperature decreased, G′ and G” increased with decreasing temperature, the elastic component of the system increased, and gel formation began. When G′ was equal to G”, the temperature at this point was the gel temperature of Ca-IC and Na-IC [44]. The solidification temperature of Ca-IC (70.9 °C) was higher than that of Na-IC (65.1 °C), which might be related to the higher gel strength of Ca-IC than that of Na-IC [45], and the molecules of Ca-IC were likely to aggregate to form a gel. Figure 8B shows the warming process. The G′ and G” values of ι-carrageenan gradually decreased with increasing temperature, indicating the process of gel network loosening [46]. Ca-IC had higher melting temperature (77.9 °C) than Na-IC (69.6 °C), indicating that ι-carrageenan (Ca-IC) extracted by the Ca(OH)_2_ method had better gel thermal stability compared to ι-carrageenan (Na-IC) extracted by the conventional alkali (NaOH) method.

#### 2.8.4. Thixotropy Test

Hysteresis loops are used to characterize thixotropy, which is evidenced by the shear stress response following a sequence of increasing shear rate scans from zero to maximum and then decreasing scans from maximum to zero [47]. As shown in Figure 8C, the shear forces of the Ca-IC and Na-IC solutions did not overlap during the rise and fall of the shear rate, forming evident thixotropic ring and indicating that both carrageenan solutions were thixotropic fluids. The thixotropic ring of the Ca-IC solution was obviously smaller than that of the Na-IC solution, which indicated that Ca-IC was less thixotropic and might be more conducive in shape retention of food in applications.

## 3. Materials and Methods

### 3.1. Materials

*E. denticulatum* was obtained from Greenfresh (Zhangzhou, China) Food Co., Ltd. Ca(OH)_2_ was obtained from Xilong Science Co., Ltd (Shantou, China). CO_2_ was obtained from Xiamen Air Separation Special Gas Industry Co., Ltd (Xiamen, China). All chemicals were of analytical grade and used directly without further purification.

### 3.2. Extraction of ι-Carrageenan by Ca(OH)_2_

Dried *E. denticulatum* (25 g) was added into water at solid–liquid ratios of 1:20, 1:25, 1:30, 1:35, and 1:40 and boiled in a water bath at 100 °C for 1 h. The mixture was added with Ca(OH)_2_ (0, 0.1%, 0.5%, 1.0%, and 1.5%, g·g^−1^), stirred thoroughly, and placed in water baths at different temperatures (60 °C, 70 °C, 80 °C, 90 °C, and 100 °C) for 2, 4, 6, 8, and 10 h. Impurities were removed using a mesh double-layer cotton filter cloth, and CO_2_ was released into the filtrate by using an air distribution tube until pH 7–8 was reached followed by a second filtration. Next, CO_2_ was passed into the extract until the filtrate became clear and transparent, and the filtrate was added into the industrial spirit at a ratio of 1:1.5 (*v*/*v*) for alcohol precipitation followed by filtration through a double-layer cotton filter cloth. Finally, after dehydration, drying at 55 °C for 12 h using the oven, and crushing steps, ι-carrageenan was obtained.

A single-factor test was performed using the above method to optimize the extraction process. Then, we experimented on a 500 L scale (Figure 2), and the carrageenan produced in the 500 L experiment was used for subsequent experiments.

### 3.3. Extraction of ι-Carrageenan by NaOH 

The commercial ι-carrageenan extracted by the NaOH method was provided by Greenfresh (Zhangzhou, China) Food Co., Ltd. and prepared as follows: NaOH–KCl mixed solutions with concentrations of 7.5% and 12.0% were prepared. About 10 kg of dried *E. denticulatum* was immersed in an NaOH–KCl solution at 43 °C at a solid–liquid ratio of 1:30 for 2.5 h. The sample was filtered and washed with 400 kg of circulating water with agitation, and the cycle was repeated thrice. The sample was stirred and washed once with 400 kg of water. Then, the sample was added into a hydrochloric acid solution at a ratio of 1:30 and acidified at a concentration of 0.35% for 0.75 h. About 800 kg of water was prepared to wash the sample to neutral. The sample was soaked in 90 °C water at a solid–liquid ratio of 1:30 for 5 h, and the filtrate was poured into industrial spirit in a ratio of 1:1.5 (*v*/*v*) to obtain a flocculent product. Finally, the product was dehydrated, air-dried with an induced draft fan at room temperature for 24–36 h, and crushed to obtain carrageenan. 

### 3.4. Yield Determination

The yield of ι-carrageenan was determined as the ratio of the weight of the final ι-carrageenan product to the weight of the dried raw material of *E. denticulatum* [19].

### 3.5. Elemental Analysis

The CHNS content was determined by combustion through the Thermo Flash 2000 Elementar Vario macro cube (Elementar, Hanau, Germany). The Ca content was determined by the ICP MS 7700ce spectrometer [19].

### 3.6. Determination of Physicochemical and Gel Properties

Gel strength was determined in accordance with the method of Yarnpakdee, Benjakul, & Kingwascharapong [16] with slight modifications. ι-carrageenan (3.0 g) was added into a 0.2% (*w*/*v*) CaCl_2_ solution to prepare a 3.0% (*w*/*v*) solution of ι-carrageenan. The solution was completely dissolved, poured into Petri dishes, and allowed to stand for 12 h. Gel strength was determined using a texture analyzer (Stable Micro System, Surrey, UK) at 25–30 °C and expressed as g·cm^−2^. 

Whiteness was measured using the WSC-C colorimeter (Precision Scientific Instruments Co., Ltd., Shanghai, China).

The sulfate and 3,6-AG contents were determined by the BaCl_2_ turbidimetric [48] and resorcinol methods [49], respectively.

Molecular weight was measured by gel chromatography by using the CTO-20A Waters 1515 (Shimadzu, Kyoto, Japan). Viscosity was then measured in accordance with the method described by Plashchina et al. [50].

### 3.7. FTIR Spectroscopy

ι-carrageenan samples were determined by a FTIR spectrophotometer (Thermo Fisher, Nicolet iS50, Waltham, MA, USA) by using the potassium bromide slice method. Carrageenan powder was mixed with KBr powder at a ratio of 1:50 in accordance with the method proposed by Miao et al. [51] and pressed into thin sheets before measurement. Scanning was performed at a wavenumber range of 4000 cm^−1^ to 500 cm^−1^.

### 3.8. Thermal Analysis

TG-DTG curves were recorded by an asynchronous thermal analyzer (SDT: Q600, TA, New Castle, DE, USA) [52]. The sample was heated in an alumina crucible within a nitrogen atmosphere. Temperatures were controlled between 20 °C and 600 °C and increased at a rate of 10 °C·min^−1^. DSC (TA: Q50, TA, SELB, Germany) was used to record the thermal changes in the powder. The sample was heated in an alumina crucible within a nitrogen atmosphere. Temperatures were controlled between 0 °C and 350 °C and increased at a rate of 12.5 °C·min^−1^.

### 3.9. TPA

About 2% and 3% (*w*/*v*) solutions of ι-carrageenan (containing 0.2% [*w*/*w*] CaCl_2_) were prepared, defoamed in a water bath at 85 °C, poured into a mold, and left to stand at 25 °C for 12 h. The gel texture properties were determined using the TA-XT plus Texture Analyzer with the following parameters: probe type, P/36R; pre/post measurement speed, 1 mm·s^−1^; test speed, 2 mm·s^−1^; deformation percentage, 50%; trigger force, 5.0 g; and compression time, 5 s.

### 3.10. Rheological Analysis

The DHR-2 rotational rheometer (TA Instruments, New Castle, DE, USA) was used to measure the rheological properties of ι-carrageenan. The type of measurement unit used for rheological testing was plate to plate.

A solution of ι-carrageenan (containing 0.2% [*w*/*w*] CaCl_2_) was prepared at concentrations of 0.2%, 0.5%, 1.0%, 1.5%, and 2.0% (*w*/*v*). In the shear mode, the temperature was fixed at 75 °C, and the shear rate was varied from 0.1 s^−1^ to 600 s^−1^.

A 1.5% (*w*/*v*) solution of carrageenan (containing 0.2% [*w*/*v*] CaCl_2_) was prepared. In the frequency scan mode, the temperature was fixed at 75 °C, and the oscillation frequency increased from 0.01 Hz to 10 Hz with a strain of 1%. In the temperature mode, the scanning speed was set to 2 °C·min^−1^, the oscillation frequency was 1 Hz, and the strain was 1%. The temperature was increased from 20 °C to 80 °C, held for 10 min, and then decreased to 20 °C.

### 3.11. Statistical Analysis

Data were analyzed using the SPSS 17.0 statistical software (IBM, Armonk, NY, USA) for Windows. All reported values were the average of at least three independent experiments, and the significance level was set at 0.05. ANOVA was used to evaluate the effect of single-factor optimization.

## 4. Conclusions

In conclusion, the extraction of ι-carrageenan by Ca(OH)_2_ is practical and feasible, with an improved gel strength and a higher product yield. Ca-IC showed no marked changes in structure and thermal stability compared to Na-IC. In terms of gel texture, Ca-IC had improved hardness and chewiness. Rheological results showed that both the Na-IC and Ca-IC solutions exhibited pseudoplastic fluid properties. In addition, the viscosity of the Ca-IC solution was lower, and the thermal stability of the gel was better compared to that of Na-IC. Therefore, this study concludes that the extraction of ι-carrageenan by Ca(OH)_2_ is industrially promising.

## Figures and Tables

**Table 1:** Physicochemical properties of Na-IC and Ca-IC.

Physicochemical Property	Na-IC	Ca-IC
Gel strength (g·cm^−2^)	235.2 ± 12.2 ^b^	337.1 ± 25.9 ^a^
Sulfate content (%)	27.0 ± 0.7 ^a^	26.7 ± 2.1 ^a^
3,6-anhydro-d-galactose content (%)	14.0 ± 0.7 ^b^	15.9 ± 0.6 ^a^
Whiteness (%)	48.6 ± 0.4 ^b^	56.7 ± 0.3 ^a^
Viscosity (cP)	50.8 ± 1.5 ^a^	25.4 ± 1.2 ^b^

Note: Different lowercase superscripts within the same column indicate significant differences (*p* < 0.05).

**Table 2:** Consistency and flow indices of ι-carrageenan extracted using different processes.

Concentration (%, *w*/*v*)	Na-IC			Ca-IC		
	K	*n*	*R* ^2^	K	*n*	*R* ^2^
0.2	0.8361	0.2139	0.9901	0.0152	0.6746	0.9944
0.5	2.7746	0.2075	0.9955	0.0548	0.6226	0.9900
1.0	5.1585	0.2032	0.9917	0.3356	0.5207	0.9953
1.5	7.8100	0.1998	0.9906	0.8052	0.5048	0.9909
2.0	8.5668	0.1822	0.9921	1.6499	0.4999	0.9905

Note: K: consistency index; *n*: flow index; *R*^2^: goodness of fit.

## Data Availability

Not applicable.

## Supplementary Materials

The following supporting information can be downloaded at: https://www.mdpi.com/article/10.3390/md20070419/s1, Figure S1: FTIR spectra (500–2000 cm^−1^); Figure S2: Carbon-13 NMR spectrum of Ca-IC and Na-IC. (G and A denote galactose and 3,6-anhydro-galactose, respectively [3,53]).
