# Supplementary material for: Eco-Friendly Extraction, Structure, and Gel Properties of ι-Carrageenan Extracted Using Ca(OH)2
Source: Mar Drugs. 2022 Jun 27;20(7):419. doi: 10.3390/md20070419 (PMC9322172; doi:10.3390/md20070419)
Supplement: Supplementary file 1 [file marinedrugs-20-00419-s001.zip › marinedrugs-1746791-supplementary.pdf]

Eco-Friendly Extraction, Structure, and Gel Properties of ι-Carrageenan Extracted Using $Ca(OH)_2$

Feng Jiang[1,2,3,+], Yao Liu[1,2,3,+], Qiong Xiao[1,2,3,4], Fuquan Chen[1,2,3,4], Huifen Weng[1,2,3,4], Jun Chen[1,2,3,4], Yonghui Zhang[1,2,3,4,*], Anfeng Xiao[1,2,3,4,*]

1 College of Food and Biological Engineering, Jimei University, Xiamen 361021, PR China; 202011832019@jmu.edu.cn (F.J.); liuyao0902@foxmail.com (Y.L.); xiaoqiong129@jmu.edu.cn (Q.X.); fqchenhy0109@jmu.edu.cn (F.-Q.C.); wenghuifen@jmu.edu.cn (H.-F.W.)；chenjun@jmu.edu.cn (J.C.);

2 National R&D Center for Red Alga Processing Technology, Xiamen 361021, PR China

3 Fujian Provincial Engineering Technology Research Center of Marine Functional Food, Xiamen 361021, PR China

4 Xiamen Key Laboratory of Marine Functional Food, Xiamen 361021, China

+ These authors contributed equally to this paper

* Correspondence: yhz@jmu.edu.cn (Y.-H.Z.), xxaaffeng@jmu.edu.cn (A.-F.X.); Tel.: +86-592-6181487 (Y.-H.Z.); +86-592-6180075 (A.-F.X.).

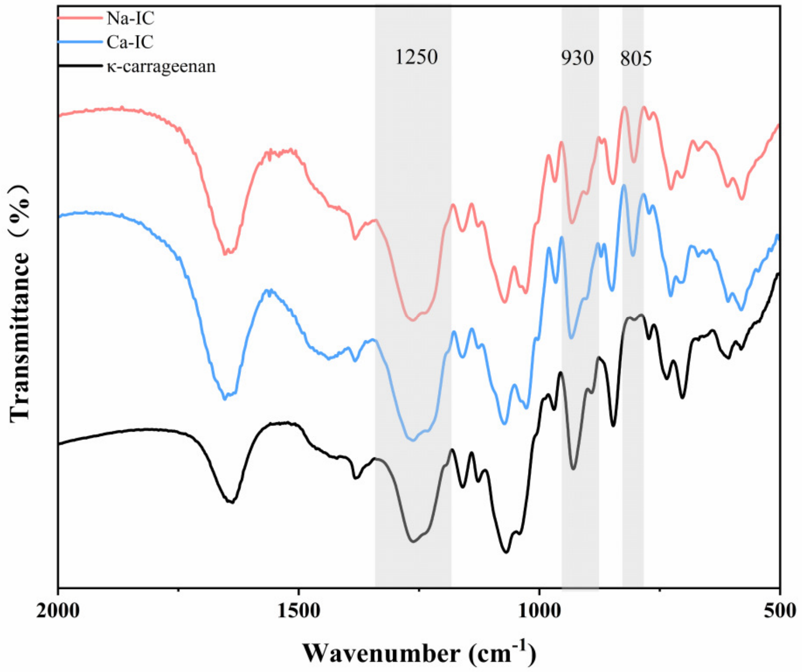

Figure S1. FTIR spectra (500-2000 cm^{-1}).

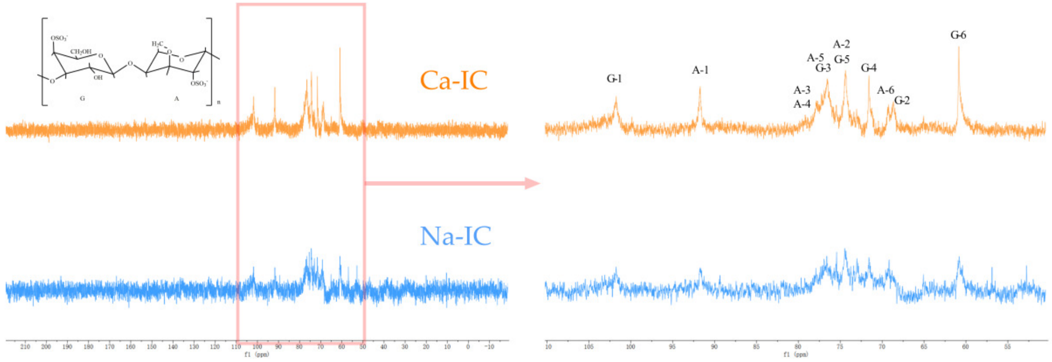

Figure S2. Carbon-13 NMR spectrum of Ca-IC and Na-IC. (G and A denote galactose and 3,6-anhydro-galactose, respectively [1, 2])

References

[1] Usov, A.; Shashkov, A., Polysaccharides of algae. XXXIV: Detection of iota-carrageenan in P*hyllophora brodiaei* (Turn.) J. Ag.(Rhodophyta) using 13C-NMR spectroscopy. **1985**.

[2] van de Velde, F.; Knutsen, S. H.; Usov, A. I.; Rollema, H. S.; Cerezo, A. S., 1H and 13C high resolution NMR spectroscopy of carrageenans: application in research and industry. *Trends in Food Science & Technology* **2002,** 13, (3), 73-92.
